# Clinical Features and Temporal Changes of RT-PCR and Chest CT in COVID-19 Pediatric Patients

**DOI:** 10.3389/fped.2020.579512

**Published:** 2020-10-09

**Authors:** Wei Xia, Yu Guo, Zhiyao Tian, Yan Luo, Daoyu Hu, Jianbo Shao, Zhen Li, Ihab R. Kamel

**Affiliations:** ^1^Department of Imaging Center, Wuhan Children's Hospital (Wuhan Maternal and Child Healthcare Hospital), Tongji Medical College, Huazhong University of Science and Technology, Wuhan, China; ^2^Department of Radiology, Tongji Hospital, Tongji Medical College, Huazhong University of Science and Technology, Wuhan, China; ^3^Russell H. Morgan Department of Radiology and Radiological Sciences, Johns Hopkins Hospital, Johns Hopkins Med Institute, Baltimore, MD, United States

**Keywords:** COVID-19, polymerase chain reaction, computer tomography, child, infection

## Abstract

**Objective:** This work aims to investigate the clinical features and the temporal changes of RT-PCR and CT in COVID-19 pediatric patients.

**Methods:** The clinical, RT-PCR, and CT features of 114 COVID-19 pediatric in-patients were retrospectively reviewed from January 21 to March 14, 2020. All patients had chest CT on admission and were identified as positive by pharyngeal swab nucleic acid test. The clinical features were analyzed, as well as the features and the temporal changes of RT-PCR and CT.

**Results:** Fever (62, 54%) and cough (61, 54%) were the most common symptoms. There were 34 (30%) cases of concurrent infections. The most common imaging features on CT were ground-glass opacities (46, 40%) and consolidation (46, 40%). The bilateral lower lobes were the most common pattern of involvement, with 63 cases (55%) involving one to two lobes, and in 32 (28%) cases CT was normal. Throughout the whole duration of COVID-19 in children, the diagnostic positive rate of RT-PCR has been far higher than that of CT (all *P* < 0.05). For RT-PCR follow-up, reliable negative results were obtained only 7 days after the onset of symptoms. Though lung involvement on chest CT progressed rapidly in several cases, lung involvement in children with COVID-19 is mild, with a median value of 2 on CT score.

**Conclusions:** RT-PCR is more reliable than CT in the initial diagnosis of pediatric patients with COVID-19. On follow-up, reliable negative RT-PCR results are available 7 days after the initial symptoms. The use of CT should be considered for follow-up purposes only if necessary.

## Introduction

Since December 2019, a newly discovered infectious disease named COVID-19, caused by a novel coronavirus (SARS-CoV-2), has wildly spread worldwide. Millions of COVID-19 patients have been confirmed, and thousands of children are also involved in this pandemic all over the world (1–3).

To date, viral nucleic acid testing and chest CT have been considered as the main diagnostic methods for patients with suspected COVID-19 pneumonia. However, in adults, the positive rate of pharyngeal swabs reverse transcription polymerase chain reaction (RT-PCR) is only 59–61.3% (4, 5). For adults, despite the possibility of false positives, the positive rate of chest CT is significantly higher than that of RT-PCR and is considered a more accurate early diagnostic tool (6–9).

For pediatric patients with suspected COVID-19, studies on chest CT values are limited (2, 10). Some studies have suggested that normal CT or lack of typical features were not uncommon in pediatric patients, especially during the early stage of the disease (11–14). The most common CT feature of pediatric patients was bilateral ground-glass opacities with a ratio of only 32.7% (11). However, the use of CT should be carefully assessed since pediatric patients are sensitive to radiation, and reducing CT scans for children is a top priority.

Compared with adults, the risk of severe or fatal COVID-19 disease is rare, while the majority of them were mild cases requiring only conventional therapy for viral pneumonia (11–13). In clinical practice, every child with fever and signs and symptoms of respiratory infection should be considered to have COVID-19, as during this pandemic healthy carriers or children with unproven COVID-19 can spread the infection to others.

In this pandemic emergency, it is important to optimize limited medical resources, reduce the radiation dose for children, and obtain a rapid and accurate diagnosis. Thus, we performed a longitudinal study to analyze the clinical characteristics, CT manifestations, and RT-PCR changes to explore early diagnosis strategies for pediatric patients.

## Materials and Methods

### Study Design and Participants

The study was performed in accordance with the Declaration of Helsinki principles and good clinical practice. The study protocol was approved by the Institutional Review Board of Wuhan Children's Hospital and Tongji Hospital, Tongji Medical College, Huazhong University of Science and Technology (Wuhan, China). Written informed consent was waived because of the emergence of this infectious disease.

A medical record review was conducted using the institution's database from January 21 to March 14, 2020 in Wuhan Children's Hospital and Tongji Hospital. A prior study including only 20 patients in Wuhan Children's Hospital only described the general clinical and CT features without follow-up and did not include the evaluation of RT-PCR (12).

The inclusion criteria were (a) age <16 years old, in-patients, (b) positive RT-PCR results of COVID-19, (c) CT in the picture archiving and communication system (PACS) was performed, and (d) discharged patients only.

The exclusion criteria were (a) CT scans performed earlier than 1 month, (b) patients with suboptimal image quality for analysis due to breathing motion artifact, and (c) unknown date of RT-PCR.

The pharyngeal swab samples of all the pediatric patients in our study were collected, and SARS-CoV-2 RNA was detected by RT-PCR. The RT-PCR kits were from Wuhan Huada Biotechnology Co., Ltd., Shanghai Huirui Biotechnology Co., Ltd., or Shanghai BioGerm Medical Biotechnology Co., Ltd. These were approved by China Food and Drug Administration for the detection of SARS-CoV-2 nucleic acid.

The discharge criteria were as follows: (1) normal temperature for at least 3 days, (2) significantly improved respiratory symptoms, and (3) two consecutive SARS-CoV-2 throat swabs with negative RT-PCR results, performed at least 24 h apart. The second result of the two consecutive RT-PCR with negative results was considered as reliable.

### Chest CT Protocols

All the CT scans, including repeated CT scans, were performed according to the clinical presentation judged by pediatricians. Non-enhanced chest CT was performed in either of the four CT units (SOMATOM Definition AS128, Siemens; uCT 780, United Imaging; Optima 660, GE; SOMATOM Definition AS+, Siemens) with the following parameters varying according to body weight: 80–120 kV, 50–120 mAs, and slice thickness of 10 mm. The scanning range covered from the lung apex to the diaphragm on axial plane taken under free breathing, with the patients in supine position. If necessary, 0.50 ml/kg body mass of 10% chloral hydrate was taken orally before the examination. Thin-section CT images were reconstructed with 0.625-mm collimation with a standard algorithm and then sent to the PACS for analysis.

### Data Collection and Analysis

We reviewed the clinical charts of all the pediatric patients for demographic information, symptoms, date of symptom onset, admission date, discharge date, and dates and results of nucleic acid tests for COVID-19 and other identified concurrent infectious pathogens.

Two radiologists (WX and ZL, with 12 and 18 years of experience, respectively) independently reviewed the chest CT images on PACS; only decisions reached in consensus were reported. The initial CT images were stratified into one of two groups: normal or abnormal groups. The CT images in the abnormal group were further assessed for imaging features including (a) unilateral or bilateral distribution, (b) lobes involved, and (c) lesion characteristics. Lesion characteristics were subcategorized into (a) ground-glass opacity, (b) consolidation, (c) nodule, and (d) thickened interlobular septa.

The involvement of the lung was quantified according to a previously published paper, which had applied it in adults with COVID-19 (15). We divided each lung into the upper zone (above the carina), the middle zone, and the lower zone (below the inferior pulmonary vein). Each zone was scored for percentage of lung involved on a scale of 0–4 (0 for 0% involvement, 1 for <25% involvement, 2 for 25% to <50% involvement, 3 for 50% to <75%, and 4 for more than 75% involvement). The overall CT score of lung involvement was the summation of scores from all six lung zones.

### Statistics

Statistical analysis was performed using SPSS 19 (IBM Corporation, NY, USA). The day of onset of symptom was set as day 0. The cumulative percentages of cases diagnosed by RT-PCR and CT as a function of time were plotted separately. Following an initial positive RT-PCR, the cumulative percentages on first negative results and final negative results of follow-up RT-PCR were plotted as well. The CT scores of lung involvement were plotted over time. The cumulative cases diagnosed by RT-PCR and CT at different time points were compared by chi-square test, two-tailed, and *P* < 0.05 was considered as a statistically significant difference, as well as for the comparison of chest CT features between cases with and without co-infection.

## Results

**Figure 1:**
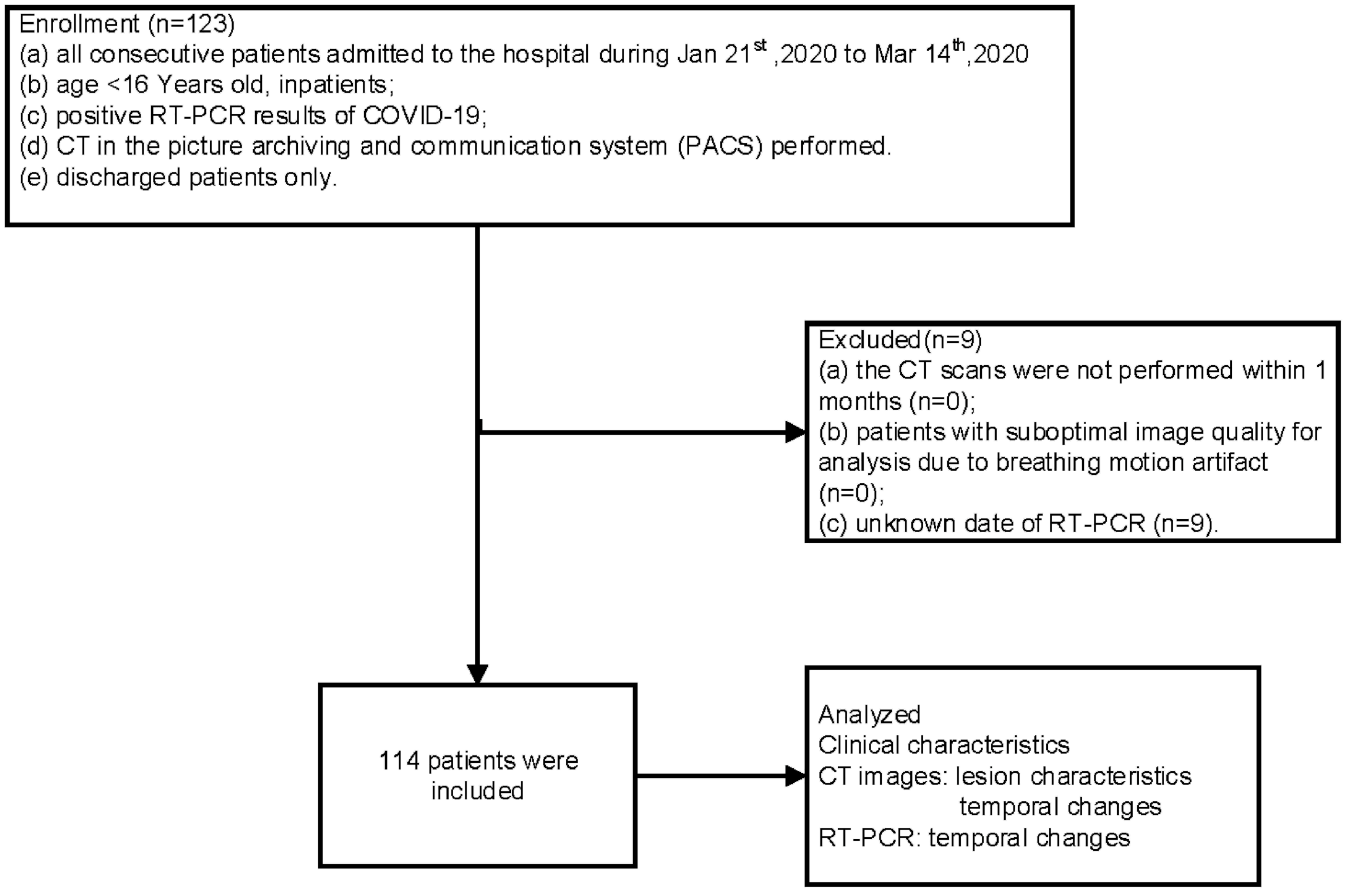
Flow chart of this study.

Between January 21 and March 14, 2020, the total number of RT-PCR confirmed COVID-19 pediatric discharged patients who have undergone CT was 123. Of those, nine cases with an unknown date of RT-PCR were excluded. The final number of patients included in this cohort was 114. All included patients were residents of Wuhan. The flow chart is shown in Figure 1.

### Clinical Characteristics

The demographics and the clinical characteristics of all the patients are summarized in Table 1. Male (69, 61%) and school-aged children (older than 6 years; 56, 49%) were more susceptible to COVID-19. In most cases, the duration from symptom onset to discharge was <21 days (87, 76%), with an average hospital stay of 13 days. Fever (62, 54%) and cough (61, 54%) were the most common symptoms. A concurrent infection was found in 34 (30%) cases, with mycoplasma (29, 25%) being the most common concurrent infectious pathogen.

**Table 1:** Demographics of the included 114 pediatric patients.

**Characteristics**	**Cases (percentage)**
**GENDER**	
Male	69 (61%)
Female	45 (39%)
**AGE**	
<6 months	11 (10%)
6 months−6 years	47 (41%)
>6 years	56 (49%)
**DURATION FROM SYMPTOM ONSET TO DISCHARGE (DAYS)**	
8–14	42 (37%)
15–21	45 (39%)
22–28	15 (13%)
>28	12 (11%)
**SYMPTOMS**	
Fever	62 (54%)
Cough	61 (54%)
Other symptoms (sore throat, diarrhea, nasal discharge, sneezing, vomiting)	42 (37%)
**CONCURRENT INFECTION**	
None	80 (70%)
One other pathogen	31 (27%)
Two other pathogens	3 (3%)

*Data are presented as cases (percentage), while percentage was calculated by the number of cases/114 cases*.

### Chest CT Features

**Figure 2:**
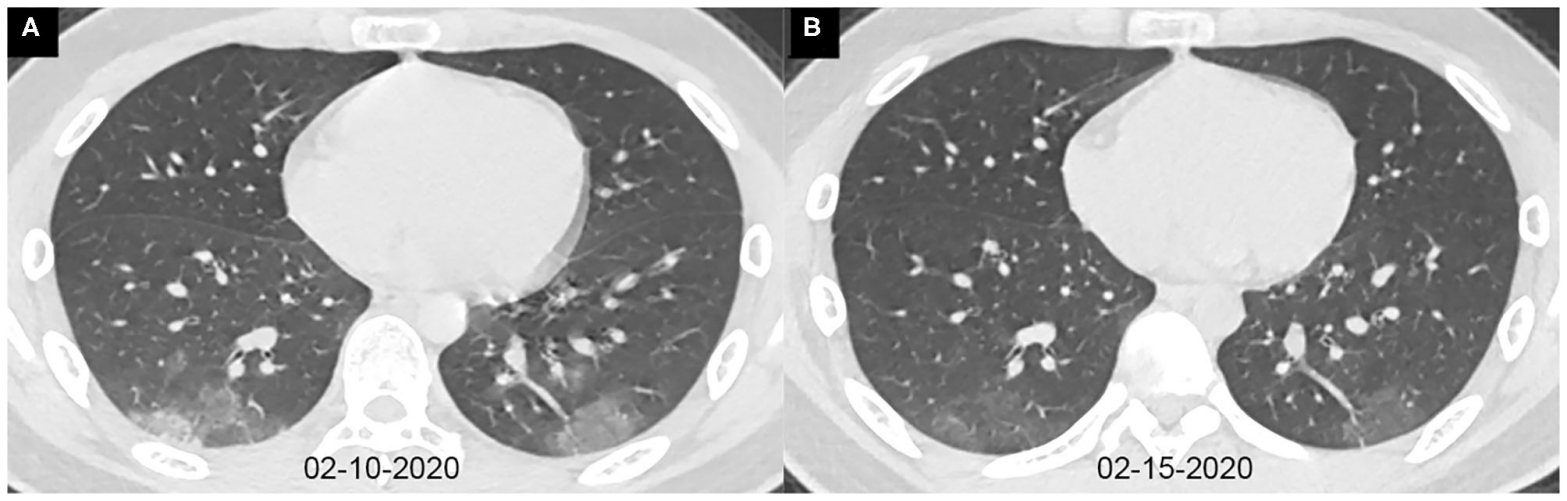
Axial chest CT images of a 13-year-old boy with fever and cough for 10 days. Pharyngeal swab RT-PCR tests were performed on February 4, 5, and 16, 2020, with positive results, and on February 20 and 21, 2020, with negative results. Chest CTs were obtained on February 10 **(A)** and 15 **(B)**. **(A)** The typical ground-glass opacities are shown in the bilateral lower lobes. **(B)** The ground-glass opacities are obviously absorbed.

**Figure 3:**
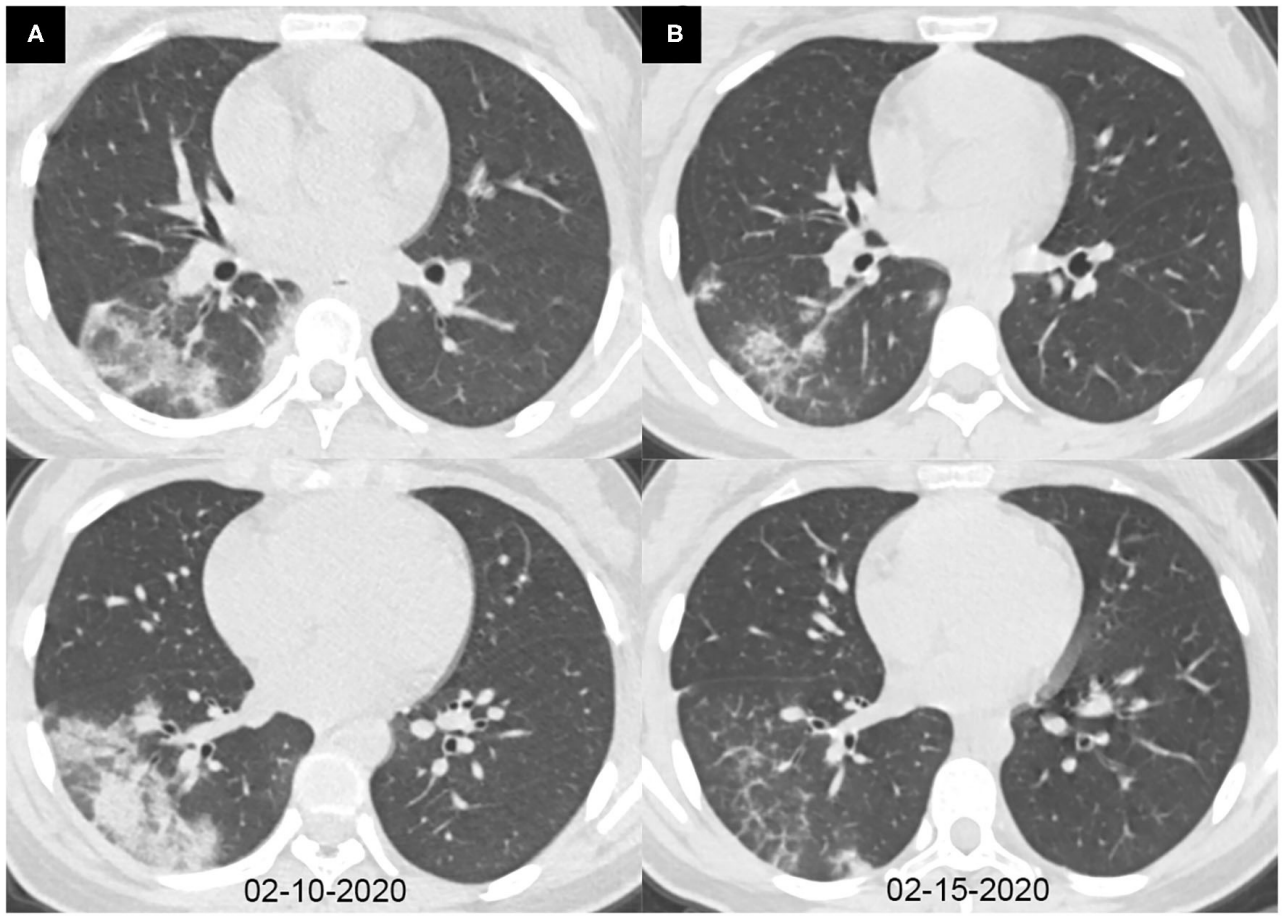
Axial chest CT images of a 14-year-old girl with fever and cough for 6 days. Pharyngeal swab RT-PCR tests were performed on February 9, 2020, with positive results, and on February 6, 13, and 15, 2020, with negative results. Chest CTs were obtained on February 10 **(A)** and 15, 2020 **(B)**. **(A)** Consolidations were seen in the right lower lobe. **(B)** The consolidations were obviously absorbed.

**Figure 4:**
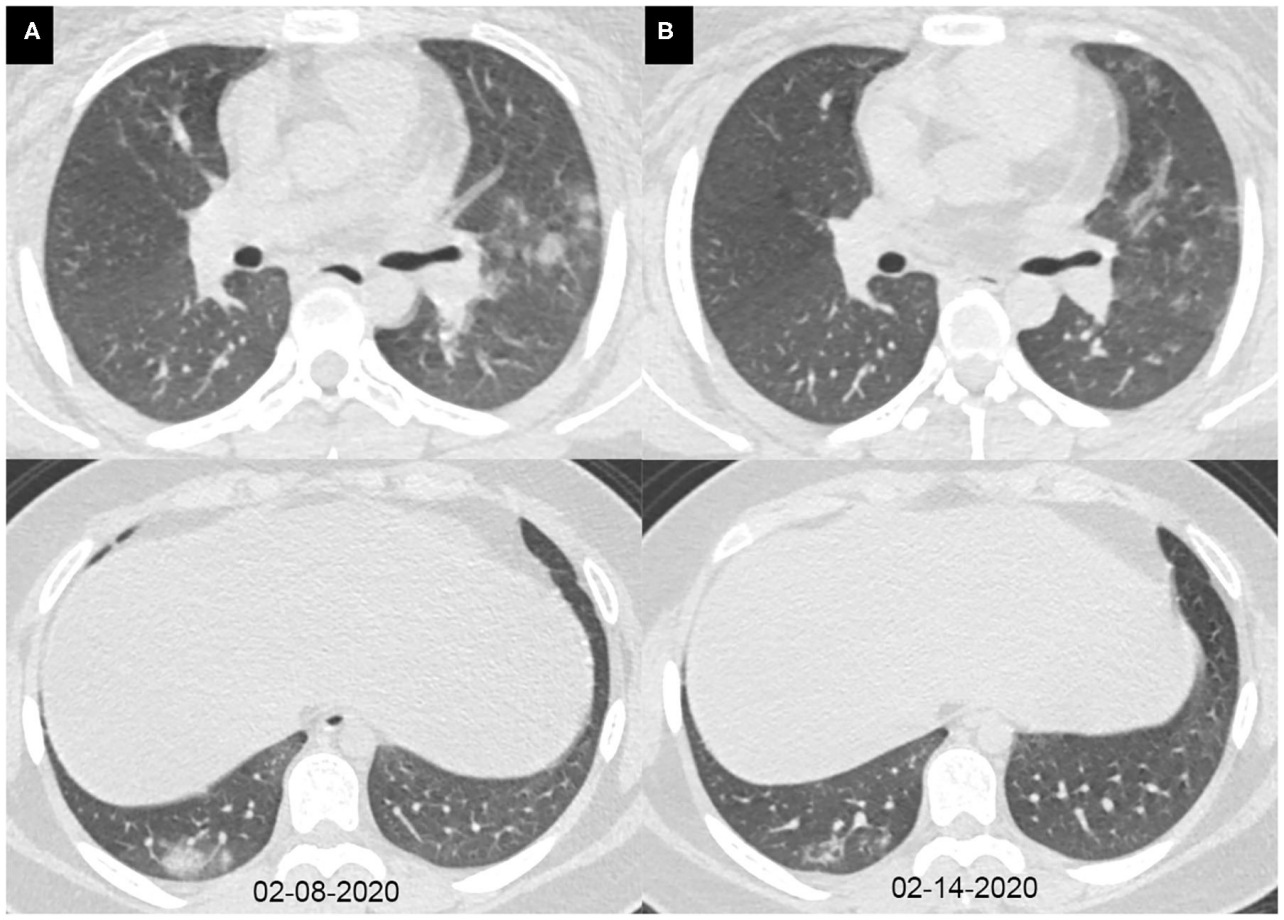
Axial chest CT images of a 15-year-old girl with fever, cough, diarrhea, and headache for 7 days. Pharyngeal swab RT-PCR tests were performed on February 12, 2020, with positive results, and on February 15 and 17, 2020, with negative results. Chest CTs were obtained on February 8 **(A)** and 14, 2020 **(B)**. **(A)** The subpleural consolidations and nodules were seen in the right lower lobe and the left upper lobe. **(B)** The consolidations and nodules were obviously absorbed.

The characteristics of chest CT are reported in Table 2. During the hospitalization period, 97 (85%) pediatric patients had no more than two chest CTs, while 17 (15%) cases had three or more CT scans, with intervals of 1 to 21 days (average, 8.9 days). Among all the patients, initially normal chest CTs were found in 32 (28%) cases, among whom 24 patients had undergone repeated CT scans. No more than two lobes were involved in 63 (55%) cases. Unilateral lung involvement was found in 46 (40%) cases, and bilateral lung involvement was found in 36 (32%) cases. The bilateral lower lobes were most susceptible to COVID-19, with 43 cases (38%) affected in both the left and the right lower lobes, respectively. Ground-glass opacity (46, 40%) and consolidation (46, 40%) were the most common lesion patterns (see Figures 2, 3 for details), while nodules were found in seven (6%) cases (see Figure 4 for details). A comparison of chest CT features between cases with and without co-infection is shown in Table 3.

**Table 2:** CT characteristics of the included 114 pediatric patients.

**CT characteristics**	**Cases (percentage)**
**NUMBER OF CHEST ct PERFORMED DURING HOSPITAL STAY**	
1	33 (29%)
2	64 (56%)
3	14 (12%)
4	3 (3%)
**INITIAL CT FINDINGS**	
Normal	32 (28%)
Abnormal	82 (72%)
Unilateral lung involvement	46 (40%)
Bilateral lung involvement	36 (32%)
**LOBAR INVOLVEMENT**	
Right upper lobe	31 (27%)
Right middle lobe	23 (20%)
Right lower lobe	43 (38%)
Left upper lobe	25 (22%)
Left lower lobe	43 (38%)
**NUMBER OF LOBES INVOLVED**	
1 lobe	33 (29%)
2 lobes	30 (26%)
3 lobes	8 (7%)
4 lobes	5 (4%)
5 lobes	6 (5%)
**LESION CHARACTERISTICS**	
Ground-glass opacity	46 (40%)
Consolidation	46 (40%)
Nodule	7 (6%)
Thickened interlobular septa	5 (4%)

*Data are presented as cases (percentage), while percentage was calculated by the number of cases/114 cases*.

**Table 3:** Comparison on CT findings between 34 patients with co-infection and 80 patients without co-infection.

**CT characteristics**	**34 cases with co-infection (percentage)**	**80 cases without co-infection (percentage)**	***P*-value**
**NUMBER OF CHEST ct PERFORMED DURING HOSPITAL STAY**			
1	9/34 (26%)	24/80 (30%)	0.704
2	19/34 (56%)	45/80 (56%)	0.971
3	6/34 (18%)	8/80 (10%)	0.409
4	0/34 (0%)	3/80 (4%)	0.614
**INITIAL CT FINDINGS**			
Normal	8/34 (24%)	24/80 (30%)	0.386
Abnormal	26/34 (76%)	56/80 (70%)	0.482
Unilateral lung involvement	12/34(35%)	34/80 (43%)	0.473
Bilateral lung involvement	14/34(41%)	22/80 (27%)	0.151
**LOBAR INVOLVEMENT**			
Right upper lobe	10/34 (29%)	21/80 (26%)	0.729
Right middle lobe	7/34 (21%)	16/80 (20%)	0.943
Right lower lobe	15/34 (44%)	28/80 (35%)	0.358
Left upper lobe	10/34 (29%)	15/80 (19%)	0.208
Left lower lobe	14/34 (41%)	29/80 (36%)	0.620
**NUMBER OF LOBES INVOLVED**			
1 lobe	8/34 (24%)	25/80 (31%)	0.406
2 lobes	13/34(38%)	17/80 (21%)	0.060
3 lobes	0/34 (0%)	8/80 (10%)	0.131
4 lobes	3/34 (9%)	2/80 (3%)	0.313
5 lobes	2/34 (6%)	4/80 (5%)	1.000
**LESION CHARACTERISTICS**			
Ground-glass opacity	14/34 (41%)	32/80 (40%)	0.907
Consolidation	15/34 (44%)	31/80 (39%)	0.593
Nodule	2/34 (6%)	5/80 (6%)	1.000
Thickened interlobular septa	2/34 (6%)	3/80 (4%)	0.993

*Data are presented as cases (percentage), while percentage was calculated by the number of cases/34 cases or number of cases/80, respectively. The P-values comparing the differences of CT findings between patients with and without co-infection are from chi-square test. P < 0.05 was considered as a statistically significant difference*.

### RT-PCR Characteristics

Detailed information on RT-PCR is displayed in Table 4. All the cases have three to 12 times RT-PCR during their hospital stay, and 89 (77%) cases have five or less RT-PCR assays, with intervals of 1–13 days (average, 3.5 days). The duration from the first positive result to the final negative result was within 21 days in 105 (92%) cases. The most common temporal tendency pattern of RT-PCR was positive to negative (91, 80%).

**Table 4:** Characteristics of RT-PCR test in 114 pediatric patients.

**Characteristics**	**Cases (percentage)**
**NUMBER OF RT-PCR TESTS PERFORMED**	
3	41 (36%)
4	28 (24%)
5	20 (17%)
6	11 (10%)
7	7 (6%)
8	3 (3%)
9	2 (2%)
10	1 (1%)
12	1 (1%)
**DURATION FROM FIRST POSITIVE TO FINAL NEGATIVE RESULT (DAYS)**	
≤ 7	21 (18%)
8–14	59 (52%)
15–21	25 (22%)
22–28	7 (6%)
>28	2 (2%)
**TEMPORAL TENDENCY OF ALL THE RESULTS (FROM SYMPTOM ONSET)**	
Positive to negative	91 (80%)
Positive to negative to positive to negative	19 (17%)
Negative to positive to negative	4 (3%)

*Data are presented as cases (percentage), while percentage was calculated by the number of cases/114 cases*.

### Temporal Changes of Initial Chest CT and RT-PCR

**Figure 5:**
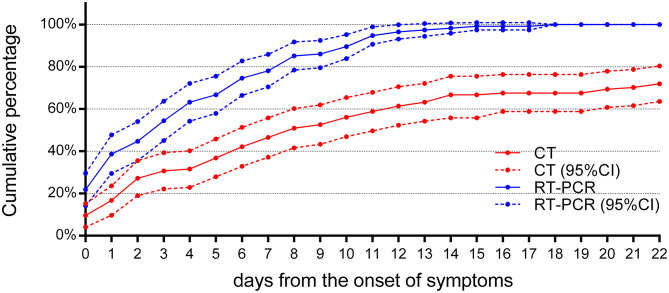
The cumulative percentage of identified cases by RT-PCR and chest CT. Both curves of RT-PCR and CT increased rapidly after the onset of symptoms (*n* = 114). By the 7th day after the onset of symptoms, 89 (78%) cases have been confirmed by RT-PCR, compared to 53 (46%) cases by chest CT (*P* < 0.05). By day 14 after the onset of symptoms, RT-PCR diagnosed 112 (98%) cases, but chest CT was positive in only 76 (67%) cases (*P* < 0.05). 95% CI, 95% confidence interval.

The cumulative percentage of cases identified by RT-PCR and initial chest CT is shown in Figure 5. In the first week after the onset of symptoms, the cumulative percentage of RT-PCR and CT both increased rapidly. On the 4th day after the onset of symptoms, 72 (63%) patients had a positive RT-PCR, but only 36 (42%) patients had infiltrates in the chest CT at the same time (χ^2^ = 22.8, *P* < 0.05). On the 7th day after the onset of symptoms, RT-PCR confirmed 89 cases (78%) of COVID-19 pneumonia, while chest CT confirmed only 53 cases (46%) (χ^2^ = 24.2, *P* < 0.05). By the 14th day of symptom onset, 112 cases (98%) were confirmed by RT-PCR, but only 76 cases (67%) were positive for chest CT (χ^2^ = 39.3, *P* < 0.05). At 18 days after the onset of symptoms, the last patient was confirmed by RT-PCR, compared to 77 (68%) identified cases by CT (χ^2^ = 44·2, *P* < 0.05). As the chest CT of 32 children was normal, the cumulative percentage of cases identified by CT was only 72% (82), compared to 114 (100%) cases identified by RT-PCR (χ^2^ = 37.2, *P* < 0.05), by day 22 after the onset of symptoms. Throughout the whole duration of COVID-19 in children, the diagnostic positive rate of RT-PCR has been far higher than that of CT (all *P* < 0.05), as shown in Table 5.

**Table 5:** Comparison of identified cases by RT-PCR and chest CT over time.

**Days from symptom onset (days)**	**Chest CT**	**RT-PCR**	***P*-value**
	**Cases (percentage, 95% CI)**	**Cases (percentage, 95% CI)**	
0	11/114 (10%, 4–15%)	25/114 (22%, 14–30%)	0.011
1	19/114 (17%, 10–24%)	44/114 (39%, 30–48%)	<0.001
2	31/114 (27%, 19–36%)	51/114 (45%, 36–54%)	0.006
3	35/114 (31%, 22–39%)	62/114 (54%, 45–64%)	<0.001
4	36/114 (32%, 23–40%)	72/114 (63%, 54–72%)	<0.001
5	42/114 (37%, 28–46%)	76/114 (67%, 58–76%)	<0.001
6	48/114 (42%, 33–51%)	85/114 (75%, 66–83%)	<0.001
7	53/114 (46%, 37–56%)	89/114 (78%, 70–86%)	<0.001
8	58/114 (51%, 42–60%)	97/114 (85%, 78–92%)	<0.001
9	60/114 (53%, 43–62%)	98/114 (86%, 80–92%)	<0.001
10	64/114 (56%, 47–65%)	102/114 (89%, 84–95%)	<0.001
11	67/114 (59%, 50–68%)	108/114 (95%, 91–99%)	<0.001
12	70/114 (61%, 52–71%)	110/114 (96%, 93–100%)	<0.001
13	72/114 (63%, 54–72%)	111/114 (97%, 94–100%)	<0.001
14	76/114 (67%, 58–76%)	112/114 (98%, 96–101%)	<0.001
15	76/114 (67%, 58–76%)	113/114 (99%, 97–101%)	<0.001
16	77/114 (68%, 59–76%)	113/114 (99%, 97–101%)	<0.001
17	77/114 (68%, 59–76%)	113/114 (99%, 97–101%)	<0.001
18	77/114 (68%, 59–76%)	114/114 (100%, 100–100%)	<0.001
19	77/114 (68%, 59–76%)	114/114 (100%, 100–100%)	<0.001
20	79/114 (69%, 61–78%)	114/114 (100%, 100–100%)	<0.001
21	80/114 (70%, 62–79%)	114/114 (100%, 100–100%)	<0.001
22	82/114 (72%, 64–80%)	114/114 (100%, 100–100%)	<0.001

*Data are presented as cases (percentage), while percentage was calculated by the number of cases/114 cases. P-values comparing the differences of cumulative diagnosed cases between RT-PCR and CT at different time points are from chi-square test. P < 0.05 was considered as a statistically significant difference. 95% CI, 95% confidence interval*.

At the same time, it was found that, after the first week of symptoms, the rise of cumulative percentage of cases identified by RT-PCR and chest CT became less and reached a plateau after 11 days. Compared with the initial chest CT, the cumulative percentage of cases identified by RT-PCR is more significant.

### Temporal Changes of RT-PCR and Chest CT

**Figure 6:**
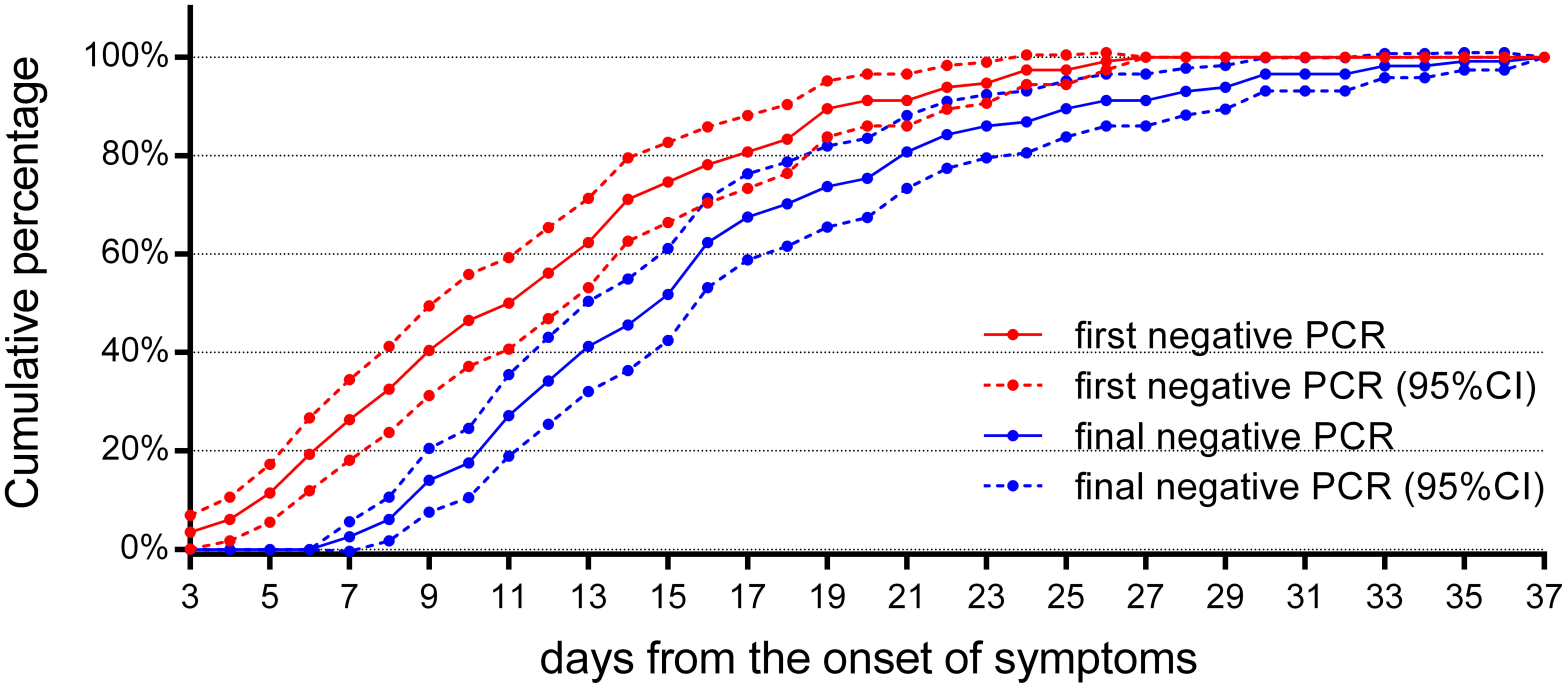
The cumulative percentages of the follow-up RT-PCR. The cumulative percentages of the first and the final negative results of follow-up RT-PCR (*n* = 114). The first negative RT-PCR results could be obtained as early as 3 days from the onset of symptoms in four cases, but they returned to positive later. A reliable final negative RT-PCR result could not be obtained until 7 days after the onset of symptoms. 95% CI, 95% confidence interval.

For treatment response, a review of the follow-up RT-PCR for children indicated that (Figure 6) there were no reliable negative RT-PCR results until 7 days after the onset of symptoms; otherwise, it should be false negative. There were 52 (46%) reliable negative RT-PCR result cases on the 14th day, 92 (81%) cases on the 21st day, and 106 (93%) cases on the 28th day, and the latest time for RT-PCR to turn negative was 37 days after the onset of symptoms.

**Figure 7:**
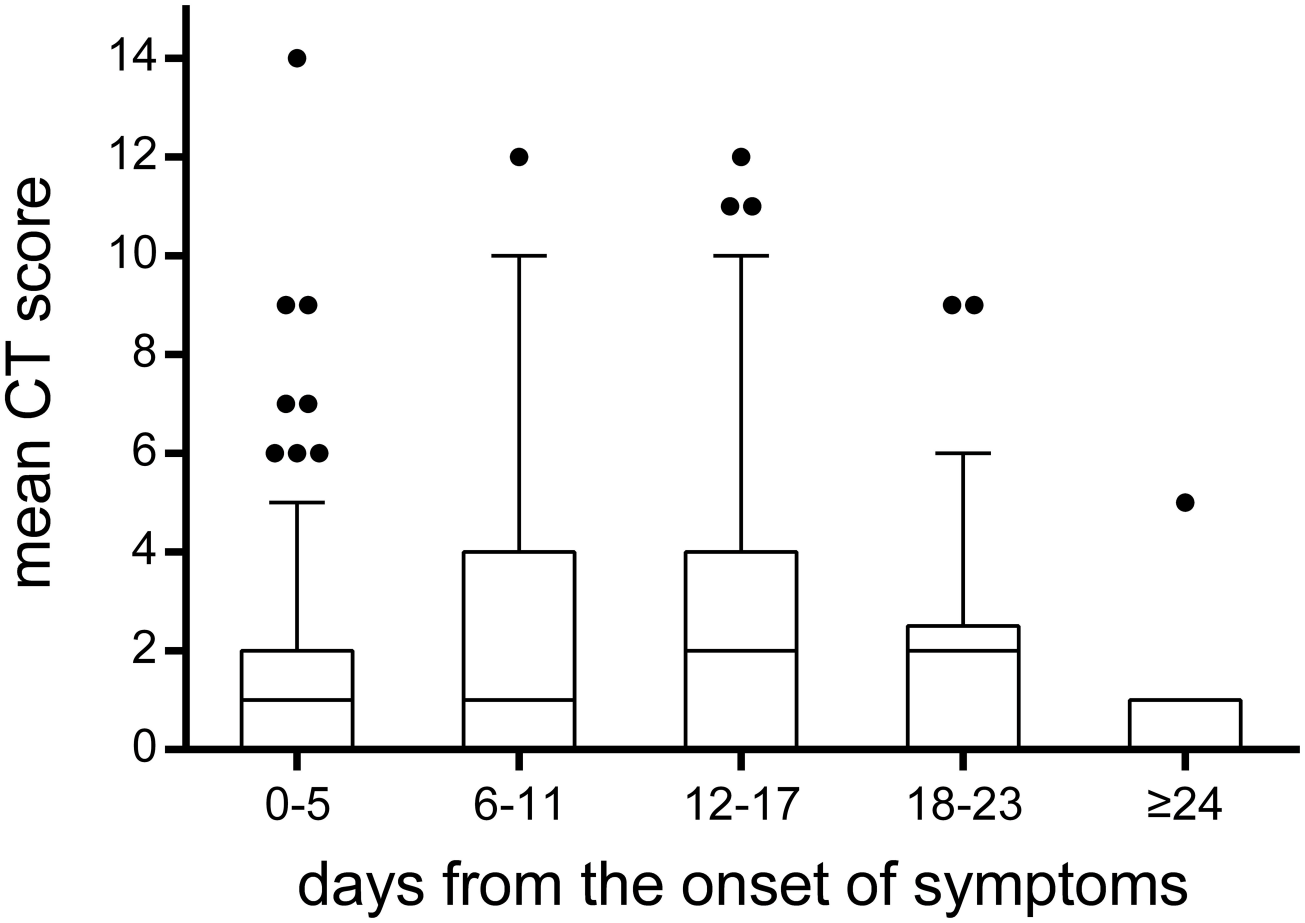
The temporal change of CT scores on lung involvement. The CT scores on lung involvement in all chest CT scans (*n* = 215). Throughout the whole duration of COVID-19 in children, the median value of CT scores was low, with a median value of 1 or 2. It was worth noting that the CT score reached 14 in one case during 0–5 days from the onset of symptoms. The bounds of the boxes represent the 25th to the 75th percentiles; the line in the middle of the box is plotted as the median. The whiskers and the outliers are drawn according to Tukey method.

A total of 215 chest CTs were obtained from 114 children with COVID-19. In Figure 7, the CT scores on lung involvement were plotted as a function of time. The CT score reached 14 in one case during 0–5 days from the onset of symptoms, but the median values of the CT scores were low before the 11th day from the onset of symptoms, with a median value of 1. The median value of CT score on lung involvement reached a peak at 12–17 days, with a median value of 2. After the 24th day from the onset of symptoms, the median value of CT score fell back to 1.

## Discussion

In the current study, the length of hospital stay in most pediatric patients with COVID-19 (average, 13 days) was not significantly different from pneumonia caused by other pathogens (average, 11.2 days) (16). Interestingly, the number of pediatric patients younger than 6 months was significantly less than that in other age groups, which may be related to residual protection from maternal immune factors, such as virus-specific antibodies according to Zeng et al. (17). The number of patients in two other age groups was not significantly different from each other. Similar to results reported in previous studies, according to lung involvement, the majority of cases were mild cases (11, 12, 18). Severe and fatal cases in pediatric patients were very rare. Thus, except for treatment for individuals, early diagnosis to avoid the further spread of the disease was an essential issue under this pandemic circumference.

In the current study, chest CT had similar characteristic manifestations including ground-glass opacities and consolidations with bilateral lower lobes, as was recently published (12, 19). Of all COVID-19-positive children in this study, completely normal chest CT was not uncommon, infiltrates on chest CT were not severe in most of them, and concurrent infections may lead to ambiguous CT imaging. Similar to previous studies, CT of the chest is often atypical, especially in the early stage, resulting in difficulty in diagnosing or ruling out COVID-19 (20). In addition, in our study, the cumulative percentage of identified cases by chest CT was low throughout the course of COVID-19, compared to RT-PCR. This is totally different from the results of related research in adults. In adults, as an important complementary tool for less-sensitive and time-consuming RT-PCR, chest CT has first been considered as a diagnostic tool for clinically confirmed cases of COVID-19 in China (21, 22). Considering that the clinical manifestations and CT features of most children are mild, CT has limited diagnostic value for children (especially 0–7 days after onset). Therefore, CT of the chest is of limited value in diagnostic algorithm and should be discouraged to reduce the radiation dose to children.

In the current study, throughout the entire course of COVID-19 pneumonia in children, the diagnostic positive rate of CT has been far lower than that of RT-PCR (all *P* < 0.05), and 28% of children have no obvious abnormal signs of CT. However, in adults, previous studies have shown that the positive rate of RT-PCR is only 59–61.3%, while the positive rate of chest CT is 88% (4, 5). These findings suggest that most infected pediatric patients have less lung involvement in the early stages of COVID-19 infection. As some COVID-19 cases confirmed by RT-PCR could have no lesions on chest CT, pathogen identification by RT-PCR has a more important role in the management of an infectious source. RT-PCR may be more reliable than CT in pediatric patients' diagnosis, and repeated RT-PCR every other day is the recommended screening for pediatric patients during the first 7 days.

In addition, the main indicator of children's discharge criteria is to determine the reliable RT-PCR negative examination results. As inappropriate sampling, preservation, and processing may lead to a low virus level, we may inevitably get false negative RT-PCR results in treatment response evaluation. Our research shows that negative RT-PCR results obtained 7 days after the onset of symptoms are reliable. This indicates that, for children whose clinical symptoms have disappeared and who may totally recover, follow-up tests of RT-PCR must be performed at least after day 7 to evaluate the efficacy.

To reduce radiation dose among the pediatric patients included in this study, most cases (85%) had one or two chest CT scans during their hospital stay. In our study, it was found that the median values of CT scores were low before the 11th day from the onset of symptoms, with a median value of 1. The median value of CT score on lung involvement reached a peak at 12–17 days, with a median value of 2. After the 24th day from onset of symptoms, the median value of CT score fell back to 1. Compared to the median CT score of 5 on lung involvement in adults reported in a previous study (15), it indicates that children with COVID-19 pneumonia are relatively mild. As reported, lung involvement peaked on 6–11 days from symptom onset in adults, while the delayed peaking in children may be related to a different immune reaction to the virus (15). In our study, the CT score reached 14 in one case during 0–5 days from the onset of symptoms, which suggested that rapid progression could also be observed in pediatric patients, even if it was rare.

There are several limitations in our study. First, even if the sample was the largest as we know, the overall cases in the two included hospitals were still limited. Second, as a retrospective study, selection bias could not be avoided. Third, during this outbreak period of COVID-19, delay in seeking care (more than 7 days from symptom onset) would influence the diagnosis and the prognosis.

In conclusion, chest CT is not recommended as a primary method for early diagnosis in children with COVID-19, especially to avoid repeated CT scans. While RT-PCR may have a more valuable position, repeated RT-PCR every other day is the recommended screening for pediatric patients during the first 7 days. For treatment response, reliable negative RT-PCR follow-up results, in accordance with discharge criteria, are not available until at least 7 days after the onset of symptoms. CT can be employed as a tool to assess lung involvement only if necessary.

## Data Availability Statement

All datasets generated for this study are included in the article/supplementary material.

## Ethics Statement

The studies involving human participants were reviewed and approved by Institutional Review Board of Wuhan Children's Hospital and Tongji Hospital, Tongji Medical College, Huazhong University of Science and Technology. Written informed consent from the participants' legal guardian/next of kin was not required to participate in this study in accordance with the national legislation and the institutional requirements.

## Author Contributions

WX, ZL, and DH conceived and designed the study. YG, YL, and DH contributed to the literature search. WX, YG, and YL collected the data. WX and YG analyzed the data. WX drafted the manuscript. ZL and IK revised the manuscript. WX and ZL had full access to all data in the study and took responsibility for the integrity of data and the accuracy of the data analysis. All authors reviewed and approved the final version of the manuscript. All authors contributed to the article and approved the submitted version.

## Conflict of Interest

The authors declare that the research was conducted in the absence of any commercial or financial relationships that could be construed as a potential conflict of interest.
